# ^18^F-FDG PET/CT versus Diagnostic Contrast-Enhanced CT for Follow-Up of Stage IV Melanoma Patients Treated by Immune Checkpoint Inhibitors: Frequency and Management of Discordances over a 3-Year Period in a University Hospital

**DOI:** 10.3390/diagnostics11071198

**Published:** 2021-07-01

**Authors:** Jean-Baptiste Le Goubey, Charline Lasnon, Ines Nakouri, Laure Césaire, Michel de Pontville, Catherine Nganoa, Diane Kottler, Nicolas Aide

**Affiliations:** 1 Dermatology Department, University Hospital, 14000 Caen, France; legoubey-jb@chu-caen.fr (J.-B.L.G.); nakouri-i@chu-caen.fr (I.N.); cesaire-l@chu-caen.fr (L.C.); depontville-m@chu-caen.fr (M.d.P.); kottler-d@chu-caen.fr (D.K.); 2 Nuclear Medicine Department, François Baclesse Cancer Centre, 14000 Caen, France; c.lasnon@baclesse.unicancer.fr; 3 INSERM ANTICIPE U 1086, Normandy University, 14000 Caen, France; 4 Nuclear Medicine Department, University Hospital, 14000 Caen, France; nganoa-c@chu-caen.fr; 5 Normandie University, 14000 Caen, France

**Keywords:** ^18^F-FDG PET/CT, contrast-enhanced CT, melanoma, metastases, tyrosine kinase inhibitors, Immune checkpoint inhibitors, follow-up, therapy monitoring

## Abstract

Aim: To perform a comprehensive analysis of discordances between contrast-enhanced CT (ceCT) and ^18^F-FDG PET/CT in the evaluation of the extra-cerebral treatment monitoring in patients with stage IV melanoma. Materials and methods: We conducted a retrospective monocentric observational study over a 3-year period in patients referred for ^18^F-FDG PET/CT and ceCT in the framework of therapy monitoring of immune checkpoint (ICIs) as of January 2017. Imaging reports were analyzed by two physicians in consensus. The anatomical site responsible for discordances, as well as induced changes in treatment were noted. Results: Eighty patients were included and 195 pairs of scans analyzed. Overall, discordances occurred in 65 cases (33%). Eighty percent of the discordances (52/65) were due to ^18^F-FDG PET/CT scans upstaging the patient. Amongst these discordances, 17/52 (33%) led to change in patient’s management, the most frequent being radiotherapy of a progressing site. ceCT represented 13/65 (20%) of discordances and induced changes in patients’ management in 2/13 cases (15%). The most frequent anatomical site involved was subcutaneous for ^18^F-FDG PET/CT findings and lung or liver for ceCT. Conclusions: Treatment monitoring with ^18^F-FDG PET/CT is more efficient than ceCT and has a greater impact in patient’s management.

## 1. Introduction

Cutaneous melanoma (CM) is an aggressive skin tumor with a high risk of visceral metastasis with a five-year relative survival rate of about 16% in metastatic cases [1]. The incidence of melanoma is increasing worldwide in white populations and is predicted to continue to increase for decades [2].

Over the last 10 years, the emergence of new therapeutics has considerably changed the prognosis of metastatic or unresectable melanoma with a marked improvement of survival compared to the era of chemotherapy [3,4,5]. Two main types of systemic treatments are now available depending on the BRAF V600 mutational status of the disease. On the one hand, a combination of targeted therapy (TT) with BRAF and MEK inhibitors can be proposed for patients with a BRAF V600 mutation-bearing tumor. On the other hand, immunotherapy (IT) with immune checkpoint inhibitors targeting antiprogrammed death 1 (PD-1) or anticytotoxic T-lymphocyte antigen 4 (CTLA-4) is proposed, single or combined, regardless of the BRAF status. These therapeutic advances have led to a profound change in the management of treatment with the possibility of several treatment lines, alone or in combination with radiotherapy, in the event of tumoral progression. Recently, ASCO proposed guidelines for the management of these systemic therapy options according to clinical parameters and BRAF mutational status [6]. Assessment of therapeutic efficacy and tolerance in metastatic patients is usually made quarterly, requiring whole-body imaging, including brain imaging, mostly assessed with brain MRI.

However, as opposed to other solid tumors, neither ESMO [7] nor ASCO [6] guidelines provide recommendations regarding which modality should be used for the extra-cerebral follow-up of metastatic melanoma patients treated with either TKIs or ICIs.

^18^F-FDG PET/CT has been proven to have high diagnostic performance for the detection of soft-tissue, nodal and visceral metastases at initial staging or during follow-up [8]. ^18^F-FDG PET/CT can identify tumor response early in the course of TKI treatment [9], for example, as early as 15 days after initiation of vemurafenib treatment [10]. In the framework of immunotherapy, ^18^F-FDG PET/CT has the unparalleled capability of assessing tumor response on a whole-body basis and detecting signs of immune activation as well as immune-related adverse effects (irAEs) [11,12,13,14,15,16,17]. However, ceCT remains the standard for therapeutic trials and may be more easily available at some centers, ensuring lower cost.

At our institution, all patients with metastatic melanoma under systemic therapy are followed up with baseline and quarterly evaluation throughout the follow-up under treatment, with a combination of contrast-enhanced CT scan (ceCT), ^18^F-FDG PET/CT, and brain MRI. ^18^F-FDG PET/CT is performed on an outpatient basis a few days before patients are hospitalized for one day to receive their treatment, the ceCT being performed during this hospitalization. The choice of this combination of imaging for extra-cerebral evaluation aims to exhaustively assess metastatic lesions. For each patient, a weekly multidisciplinary consultation meeting analyzed the quarterly assessment in order to decide on the continuation of treatment.

The aim of the present observational study was to perform a comprehensive analysis of discordances in the treatment response extra-cerebral evaluation of stage IV melanoma patients when using a combination of ceCT and ^18^F-FDG PET/CT, including the anatomical site(s) of discordance and the change(s) in patients’ management induced by these discordances.

## 2. Materials and Methods

### 2.1. Study Design

**Figure 1:**
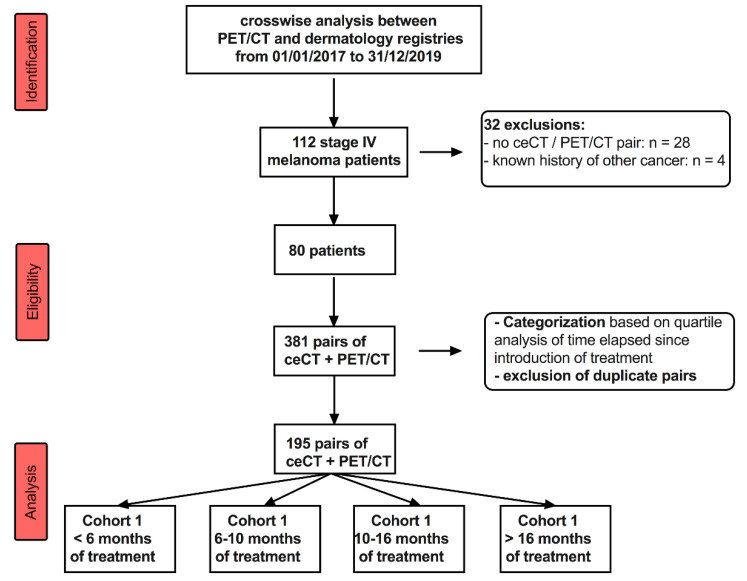
Consort diagram defining the study population.

We conducted a retrospective monocentric observational study over a 3-years period in metastatic or unresectable melanoma patients aged over 18, and who were referred for ^18^F-FDG PET/CT and ceCT, in the framework of extra-cerebral therapy monitoring of ICIs (Figure 1). Inclusion criteria were: (i) stage IV melanoma patients receiving ICIs, (ii) availability of baseline ^18^F-FDG PET/CT and ceCT pair before systemic treatment, and (iii) first ^18^F-FDG PET/CT and ceCT treatment monitoring performed between 1 January 2017 and 31 December 2019. Institutional review board approval was obtained (ref CLERS 1690) and waived the need for informed signed consent. In accordance with the European General Data Protection Regulation, we sought approval to collect data for this work from the national committee for data privacy, with the registration no. 2081250 v 0.

### 2.2. ^18^F-FDG PET/CT Protocol

Patient preparation in the PET unit and PET acquisition and reconstructions was performed as per the European Association of Nuclear Medicine (EANM) guidelines for PET tumor imaging [18], our PET unit being EANM research Ltd. (EARL) accredited since 2015 [19,20]. ^18^F-FDG was injected after the glucose level had been checked to be <200 mg/dL in patients who had been fasting for at least 4 h. Patients were provisionally scanned 60 min after the tracer injection. They were scanned from the base of the skull to mid-thigh with the arms on their sides for upper limb melanoma patients, or whole-body scanned for patients with primary melanoma of the lower limb or in patients with known distal subcutaneous metastases.

Two different PET/CT scanners were used: a Vereos system (Philips Medical Systems. Cleveland OH) and a Biograph TrueV with extended field-of-view (Siemens Medical Solutions, Erlangen, Germany). Details regarding acquisition and reconstruction parameters can be found elsewhere [21].

### 2.3. Diagnostic CT Scan

ceCT scans were performed at our institution according to local protocol involving injection of contrast media, except in the case of contraindication, followed by exploration of the chest and the abdomen.

### 2.4. Extraction and Quotation of ^18^F-FDG PET/CT and ceCT Reports

^18^F-FDG PET/CT and ceCT reports were extracted from the patients’ medical records and analyzed by two physicians in consensus. For patients with dissociated findings, i.e., patients with a mix of responding and non-responding target lesions, ^18^F-FDG PET/CT or CT examinations were reviewed on a dedicated workstation and clinical benefit was evaluated based on the tumor burden of progressing versus non-progressing lesions.

Examinations were finally classified as follows:

- with a clinical benefit: complete response, partial response, stable disease.
- with no clinical benefit: progressive disease.
- inconclusive

### 2.5. Analysis of Discordant Findings between ^18^F-FDG PET/CT and ceCT Scans

Whenever a discordance was observed between ^18^F-FDG PET/CT and ceCT reports, the anatomical site responsible for this discordance was noted, and conclusions of the multidisciplinary staff meeting discussing this discordance were noted and categorized as follows:

- Biopsy of one of the anatomical sites/surgery;
- Complementary radiological examination (such as MRI or echography);
- No change, follow-up;
- Switch from one line of treatment to another;
- Radiotherapy.

### 2.6. Statistical Analysis

Quantitative variables are presented as mean (SD).

Quartiles of the evaluation time from treatment initiation were used to classify examinations as follows:

(i). early assessment: <6 months,
(ii). interim assessments: 6–10 months and 10–16 months, and
(iii). late assessments, >16 months.

One examination per patient and per time point was kept. In the case of patients’ multiple examinations per time frame, only the earliest was considered.

Concordance between ceCT and ^18^F-FDG PET/CT reports were evaluated using the Cohen’s kappa and the reported Kappa values were classified according to the Landis and Koch benchmark, as follows:

- 0.0–0.20: poor agreement;
- 0.21–0.40: fair agreement;
- 0.41–0.60: moderate agreement;
- 0.61–0.80: good agreement;
- 0.81–1.00: very good agreement.

Fischer tests were used to seek associations between histoprognostic characteristics and the occurrence of discordances between ceCT vs. 18 F-FDG PET/CT.

For all statistical tests, a two-tailed P value of less than 0.05 was considered statistically significant. Graphs and statistical analysis were performed on XLSTAT Software (XLSTAT 2017: Data Analysis and Solution for Microsoft Excel, Addinsoft, Paris, France (2017)).

## 3. Results

### 3.1. Patients’ Demographics

After searching our database, out of 132 patients screened, 80 patients met the criteria and were included. A detailed flow chart of patients’ inclusion can be seen in Figure 1. The mean age at diagnosis was 61 years (range: 23–89 years). Nodular melanoma and superficial spreading melanoma were the two most frequent subtypes, accounting for 20 and 37.5%, respectively. BRAF^V600^ mutation was found in 34 patients (42.5%). Patients’ characteristics and histopronostic variables from the primary lesion are displayed in Table 1.

### 3.2. ^18^F-FDG PET/CT and ceCT Scans

A total of 195 pairs was analyzed. Mean (SD) time between each pair of ^18^F-FDG PET/CT and ceCT examinations was 10 (9.7) days. ^18^F-FDG PET/CT were always performed prior to ceCT.

^18^F-FDG PET/CT scans were quoted as complete metabolic response (CMR), PMR (partial metabolic response), SMD (stable metabolic disease), and progressive metabolic disease (PMD) in 25.6, 20.0, 6.2, and 43.6%, respectively. No inconclusive report was noted. Dissociated responses occurred in 4.6%.

**Figure 2:**
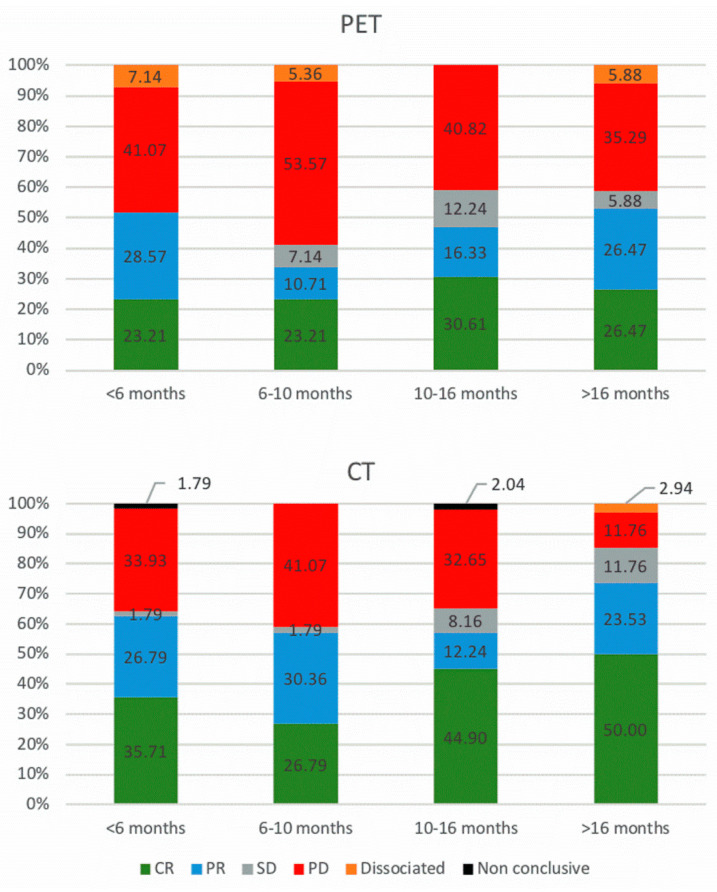
Repartition of imaging response for ^18^F-FDG PET/CT (upper panels) and ceCT (lower panels), categorized based on the time elapsed since introduction of treatment (defined as quartiles). CMR, complete metabolic response; PMR, partial metabolic response; SMD, stable metabolic disease; PMD, progressive metabolic disease; CR, complete response; PR, partial response; SD, stable disease; PD, progressive disease. n = 56, 56, 49 and 34 for <6 months, 6–10 months, 10–16 months and >16 months evaluations, respectively.

**Figure 3:**
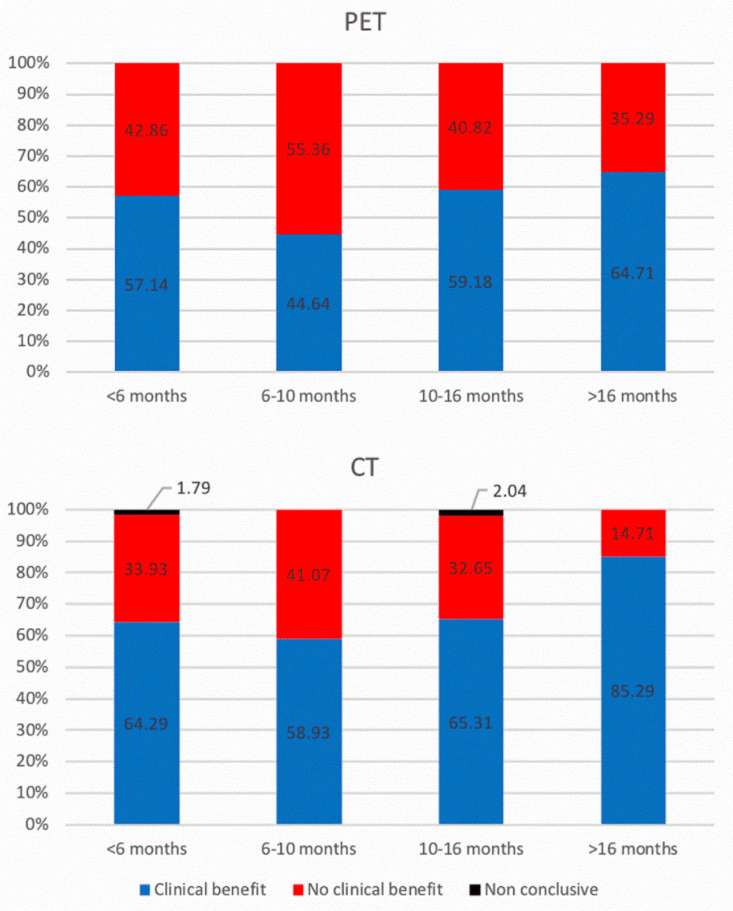
Repartition of imaging response for ^18^ F-FDG PET/CT (upper panels) and ceCT (lower panels), categorized based on the time elapsed since introduction of treatment (defined as quartiles). Patients with a clinical benefit: complete response, partial response, stable disease. Patients with no clinical benefit: progressive disease. n = 56, 56, 49 and 34 for <6 months, 6–10 months, 10–16 months and >16 months evaluations, respectively.

ceCT scans were quoted as complete response (CR), partial response (PR), stable disease (SD), and progressive disease (PD) in 37.9, 23.6, 5.1, and 31.8%, respectively, and were considered as inconclusive in 1.0%. Dissociated responses occurred in 0.5%. Figure 2 displays the repartition of responses for ^18^F-FDG PET/CT scans and ceCT. Figure 3 displays the repartition of responses for ^18^F-FDG PET/CT scans and ceCT when grouping responses based on clinical benefit.

Concordance between CeCT vs. ^18^ F-FDG PET/CT for treatment response classification was fair or moderate. When categorizing responses based on the clinical benefit, agreement between ceCT and ^18^F-FDG PET/CT was good, except for the early interim and late evaluations where it was moderate and fair, respectively. Table 2 displays kappa values in detail.

### 3.3. Timeline, Causes, and Consequences of Discordances

**Figure 4:**
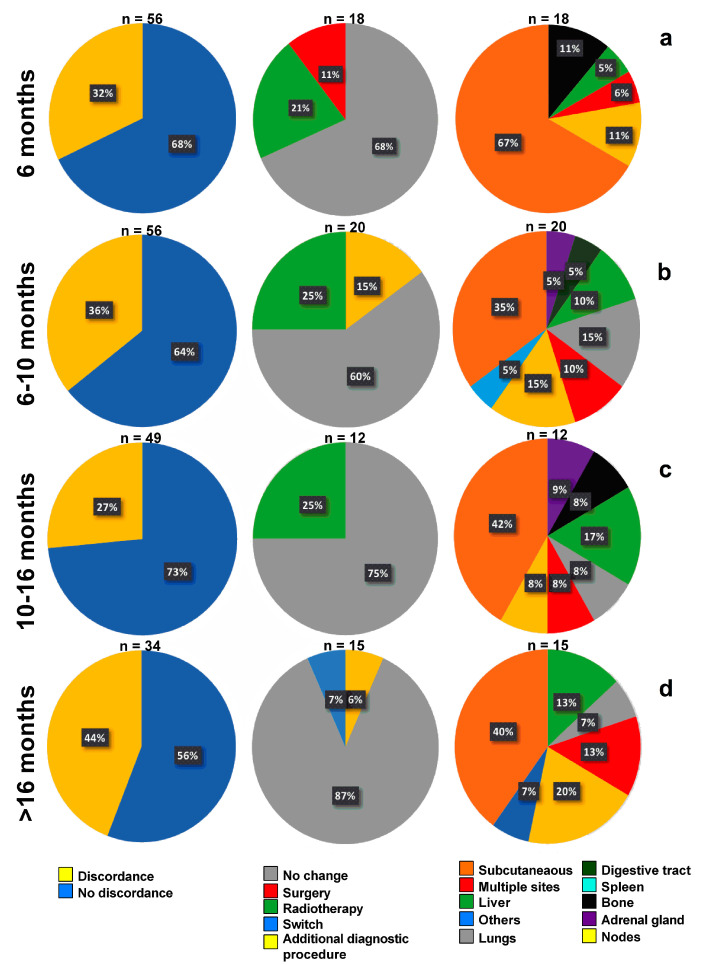
From left to right: repartition of discordance in imaging response between ^18^ F-FDG PET/CT and ceCT, induced changes in patient’s management, and anatomical site responsible for the observed discordances. Data are categorized based on the time elapsed since introduction of treatment (defined as quartiles) (**a**) early evaluation, (**b**,**c**) interim evaluation, and (**d**) late evaluation.

Overall, discordances occurred in 65 cases (33%). When categorizing imaging based on the duration of treatment, discordances occurred in around a third of patients scanned for early therapy assessment (Figure 4a), early interim evaluation (Figure 4b) and late interim evaluations (Figure 4c) while it increased to 44% for late evaluation (Figure 4d).

When grouping categories of responses into two main categories (clinical benefit vs. no clinical benefit), the number of discordances decreased from 65 (33%) to 38 (19%).

The main anatomical site of discordances between ^18^F-FDG PET/CT and ceCT scans were subcutaneous metastases, with a peak during early evaluation where this site represented 67% of discordances (Figure 4a). It was followed by nodes, with a peak (20%) during late evaluation (Figure 4d) and liver responsible for at least 10% of discordances during interim and late evaluations (Figure 4b–d).

Discordances between ^18^F-FDG PET/CT and ceCT scans were followed by no change in patient management in around two-thirds of cases during the earliest phase of treatment (Figure 4a–c), with an increase at 75 and 80% during late assessments (Figure 4d). In those cases, patients went on with the usual quarterly evaluation.

Neither histoprognostic variables, including BRAF status nor location of the primary lesion were associated with the occurrence of discordances between ceCT and ^18^F-FDG PET/CT (Supplemental Table S1).

### 3.4. Impact of Discordances on Patient’s Management

Most of the discordances (52/65, 80%) were due to ^18^F-FDG PET/CT scans upstaging the patient. Amongst these PET-related discordances, 17/52 (33%) led to change in patient’s management, the most frequent being radiotherapy of a progressing site. Switch from one line of treatment to another occurred only in one case during the late phase of treatment.

ceCT represented 13/65 (20%) of discordances and as opposed to ^18^F-FDG PET/CT. ceCT-induced changes in patients’ management were fewer (2/13, 15%).

**Figure 5:**
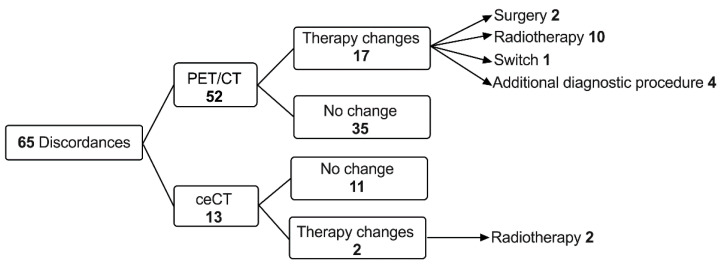
Flowchart of changes in patient management related to discordances between ^18^F-FDG PET/CT and ceCT scans.

Details regarding patient management can be found in Figure 5.

**Figure 6:**
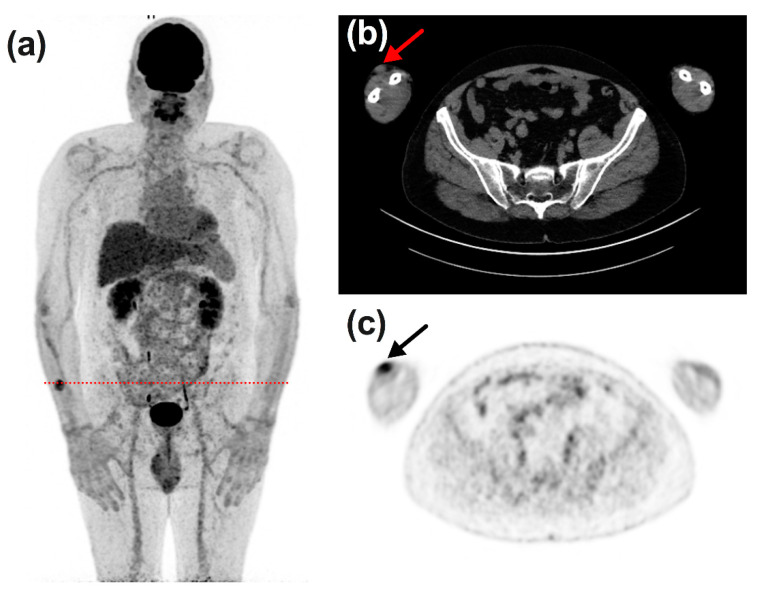
65-year-old male patient diagnosed with stage IV trunk melanoma (Breslow 0.7 mm, BRAF+) and treated with nivolumab. ^18^F-FDG PET/CT—(**a**), maximum intensity view; (**b**) CT transverse slice; (**c**) PET transverse slice} depicted progression of a subcutaneous nodule after 20 cycles of treatment, while ceCT determined stable disease. This patient was treated by radiotherapy.

**Figure 7:**
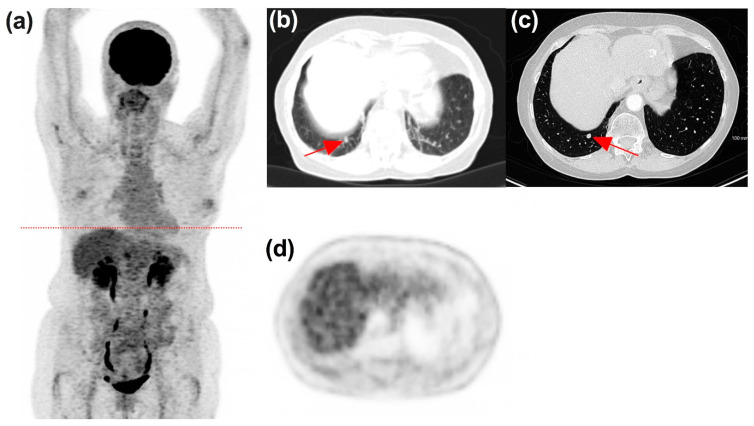
60-year-old female patient diagnosed with stage IV choroidal melanoma (BRAF-) treated with nivolumab. ceCT— (**a**), maximum intensity view; (**b**), low-dose CT from the PET/CT scan transverse slice; (**c**) diagnostic CT transverse slice; (**d**) PET transverse slice depicted progression of one pulmonary nodule after 3 cycles of treatment, while ^18^F-FDG PET/CT determined stable disease. Note the nodule overlooked on low-dose CT (red arrow, (**b**)) and not ^18^F-FDG avid (**d**), and well seen on diagnostic CT (red arrow, (**c**)).

**Figure 8:**
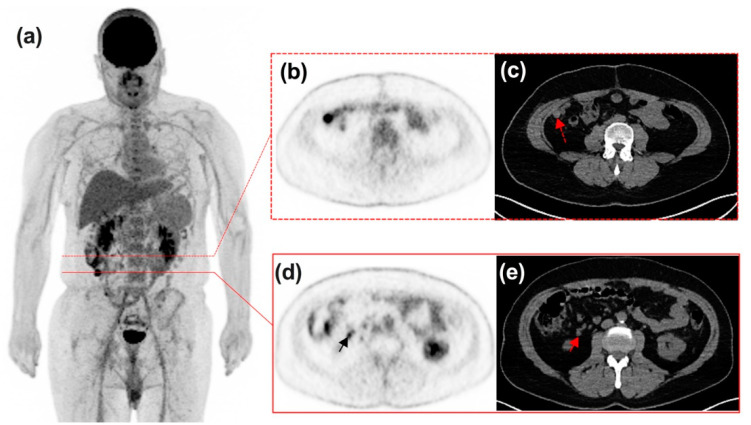
50-year-old male patient diagnosed with stage IV melanoma of lower limbs (Breslow 0.6 mm, BRAF+) and treated with TKI. ^18^F-FDG PET/CT—((**a**) maximum intensity view; (**b**,**d**) PET transverse slice; (**c**,**e**) CT transverse slice) depicted progression of carcinoma nodules after 19 months of treatment (red dotted arrow, red and black arrows), while ceCT determined stable disease. This patient was switched to nivolumab.

**Figure 9:**
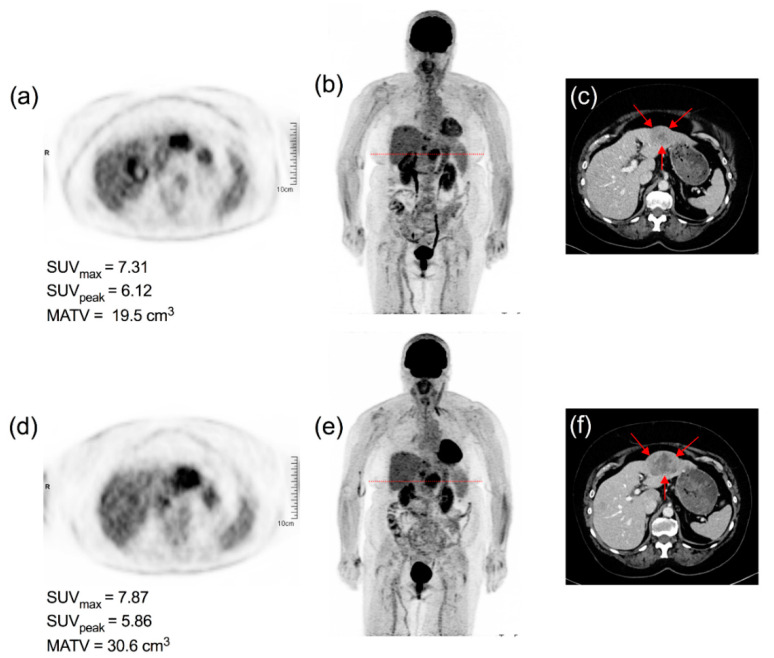
65-year-old female patient diagnosed with stage IV choroidal melanoma and treated with pembrolizumab (BRAF-). ^18^F-FDG PET/CT—(**b**,**e**) PET transverse slice at the level of the liver target lesion (red dotted line); (**a**,**d**) PET maximum intensity view} and corresponding CT transverse slice (**c**,**f**) are shown. ceCT classified this patient as progressive based on RECIST 1 dimensions of the target lesion (red arrows), while PET considered this as a stable metabolic disease based on the stability of tumor intensity. Immunotherapy was not withdrawn because of the lack of efficient second-line therapy.

Figure 6, Figure 7, Figure 8 and Figure 9 display representative examples of PET- or ceCT-related discordances.

It is noteworthy that despite the stability of tumor ^18^F-FDG uptake, the target lesion also displayed a significant increase in tumor metabolic, active tumor volume (MATV) and should therefore had been classified as progressive disease if PERCIST criteria [22] had been applied.

## 4. Discussion

While the therapeutic strategy is codified by recent guidelines [6,7], treatment monitoring of patients with melanoma remains at the discretion of clinicians and the availability of imaging. Numerous studies have shown the performance of PET in the staging of patients with melanoma [8,23,24], but few have discussed the added value of its use in treatment monitoring of ICIs [13,25] in clinical routine and CeCT continues to be the gold standard in trials to assess extra-cerebral response.

Our study involved eighty patients and 195 pairs of ^18^F-FDG PET/CT and ceCT scans. Overall, discordances occurred in 65 cases (33%). It is noteworthy that the number of screened pairs was higher (n = 381, see CONSORT flowchart on Figure 1), but we categorized patients referred for early assessment versus those referred for interim or late assessment, and excluded duplicate or triplicate pairs, leading to the final number of 195 pairs of ^18^F-FDG PET/CT and ceCT scans.

The findings from our study are 4-fold: (i) most of the observed discordances (80%) were related to ^18^F-FDG PET/CT findings, and a third of these discordances led to a change in patient’s management; (ii) neither histoprognostic variables nor location of the primary lesion were able to predict the occurrence of discordances between ceCT and ^18^F-FDG PET/CT; (iii) ceCT led to fewer discordances and changes in patient’s management were scarce; (iv) the more frequent anatomical site involved was subcutaneous for ^18^F-FDG PET/CT and lung or liver for ceCT. The latter point is due to the fact that subcutaneous lesions are easier to spot on ^18^F-FDG PET/CT and are often overlooked by CT or are even not part of the regions explored by CT when they are located on the limbs, as shown on Figure 6. The superiority of ceCT is linked to the choice for many PET centres to use low-dose CT, i.e., to perform CT only for attenuation correction and localization purposes. These low-dose CT are not adapted to the detection of small lung nodules. Finally, it should be noted that the readings of ceCT and ^18^F-FDG PET/CT are not supposed to be influenced by the age of the patients. Therefore, its influence on the occurrences of discrepancies has not been specifically studied here. However, age descriptive data in our series are representative of previous epidemiological reports [26].

Several reports have highlighted the increasing cost of treating melanoma, being driven by an increased incidence of the disease and by the introduction of expensive drugs [27,28,29]. For example, a recent study evaluating the cost of immunotherapies and targeted therapies in metastatic melanoma across 26 centers reported a cost multiplied by 104 since 2004 in France, drugs representing 80% of the total cost [28]. The high cost of treating advanced-stage melanoma obviously warrants the need to promote prevention and early detection, but also to optimize the use of systematic treatment, the latter requiring an appropriate use of imaging procedures for follow-up and treatment response evaluation. Indeed, in addition to drug cost, other costs such as extensive laboratory and imaging procedures have to be considered. An early diagnosis of progression will, in theory, allow withdrawal of an expensive therapy. In this study, therapeutic modifications consisted mostly of adding radiotherapy to non-responder metastatic sites. This management is supported by the search for an abscopal effect in the event of immunotherapy and the maintenance of a line of treatment [30].

Based on the findings from the present study, we have decided to modify our ^18^F-FDG PET/CT protocol that now includes an unenhanced lung diagnostic CT scan (acquired in deep inspiration and breath-hold), and to stop systematically performing ceCT, except in case contrast enhancement is required, such as for planning surgery. By proceeding this way, whole-body ^18^F-FDG PET/CT and brain MRI fully cover the metastatic spread patterns of melanoma.

In order to recommend this practice to other centers, a prospective study is required. The design of such a study is likely to depend on which imaging test is the standard of care in a given center: in a center using ceCT as a standard, (1) PET/CT and ceCT would be blinded to each other, (2) planned patient’s management based on ceCT should be decided, and (3) a potential change in patient management based on PET/CT would then be prospectively recorded during tumor boards using standardized questionnaires [31,32] inquiring if and how PET/CT findings altered patient’s stage and their clinical management decisions. Ideally, (4) the relevance of induced changes should be evaluated.

When it comes to the few liver metastases overlooked by ^18^F-FDG PET/CT, it is expected that advances in PET technology such as digital PET will improve the detectability of such lesions [33]. Moreover, in addition to its capability to perform a whole-body assessment of disease extension, ^18^F-FDG PET/CT is able to detect signs of immune activation with excellent reproducibility [16] and relevant immune-related adverse events, which may precede clinical diagnosis [12].

This study has several limitations. First, it was retrospective and the relevance of changes induced by imaging could not be assessed. However, the series of patients was extracted from a crosswise analysis between the Dermatology and Nuclear Medicine departments over a 3-years period and is therefore exhaustive. In addition, we did not stratify our results based on the line or on the type of treatment. The fact that imaging reports were extracted from the patients’ medical records may be regarded as a bias, as radiologists may have had access to PET reports when interpreting ceCT, and vice versa. However, it is noteworthy that because of the patient’s management at our institution, patients receiving immunotherapy always had their PET/CT scan performed prior to the ceCT. Finally, the patient’s management in terms of rhythm for follow-up at our center does not necessarily reflect the situation at other centers. Although not being a limitation, it is noteworthy that the problem of using ceCT in addition to ^18^F-FDG PET/CT does obviously not apply to centers where a “one-stop-shop” ^18^F-FDG PET/CT examination is performed using contrast enhancement for CT [34].

## Figures and Tables

**Table 1:** Patients’ characteristics and histopronostic variables from the primary lesion.

Variable\Statistic	Categories	Frequency	Relative Frequency (%)
Gender	Female	48	60.0
	Male	32	40.0
Location	Lower limb	20	25
	Upper limb	16	20
	Trunk	15	18.8
	Head and neck	11	13.7
	No primary lesion	11	13.7
	Others	7	8.8
Stage at diagnostic	IA	4	5.0
	IB	7	8.8
	IIA	6	7.5
	IIB	16	20.0
	IIC	6	7.5
	IIIB	2	2.5
	IIIC	1	1.3
	IIID	4	5.0
	IV	15	18.8
	na	7	8.8
	Missing	12	15.0
Histology	Acral lentiginous melanoma	2	2.5
	Lentigo malignant melanoma	1	1.3
	Nodular melanoma	16	20.0
	Superficial spreading melanoma	30	37.5
	Others	9	11.3
	No primary lesion	11	13.8
	Missing	11	13.8
Breslow	in situ	1	1.3
	0.1–1	7	8.8
	1.01–2	13	16.3
	>2	35	43.8
	na	18	22.5
	Missing	6	7.5
Ulceration	No	23	28.8
	Yes	26	32.5
	na	18	22.5
	Missing	13	16.3
Regression	No	40	50.0
	Yes	4	5.0
	na	18	22.5
	Missing	18	22.5
Mitotic index	High	16	20.0
	Low	8	10.0
	na	18	22.5
	Missing	38	47.5
BRAF mutation	Yes	34	42.5
	No	46	57.5

**Table 2:** Concordance between CeCT vs. ^18^ F-FDG PET/CT for treatment response classification.

	Concordance (Cohen’s Kappa)			
	<6 Months	6–10 Months	10–16 Months	>16 Months
Clinical benefit * vs. no clinical benefit **	0.71	0.51	0.67	0.39
CR vs. SD vs. PR Vs. PD vs. dissociated response	0.32	0.28	0.51	0.57

CR, complete response; SD, stable disease; PR, partial response; PD, progressive disease. * patients with clinical benefit: complete response, partial response, stable disease; ** patients with no clinical benefit: progressive disease.

## Data Availability

Not appliable.

## Supplementary Materials

The following are available online at https://www.mdpi.com/article/10.3390/diagnostics11071198/s1, Table S1: Impact of histoprognostic characteristics on the occurrence of discordance between ceCT Vs 18 F-FDG PET/CT for treatment response classification.
