# Supplementary material for: 18F-FDG PET/CT versus Diagnostic Contrast-Enhanced CT for Follow-Up of Stage IV Melanoma Patients Treated by Immune Checkpoint Inhibitors: Frequency and Management of Discordances over a 3-Year Period in a University Hospital
Source: Diagnostics (Basel). 2021 Jul 1;11(7):1198. doi: 10.3390/diagnostics11071198 (PMC8304093; doi:10.3390/diagnostics11071198)
Supplement: Supplementary file 1 [file diagnostics-11-01198-s001.zip › diagnostics-1253647-supplementary.pdf]

Table S1. Impact of histoprognostic characteristics on the occurrence of discordance between ceCT Vs ^{18}F-FDG PET/CT for treatment response classification

Variables		<6 months		6 to 10 months		10 to 16 months		>16 months	
		Numbers of observation/ discordances	*p* value	Numbers of observation/ discordances	*p* value	Numbers of observation/ discordances	*p* value	Numbers of observation/ discordances	*p* value
**Location**	Head/Neck/Trunk<br>Lower limbs<br>Upper limbs<br>Others	55 / 18	0.500	56 / 20	0.600	48 / 13	0.970	34 / 15	0.688
**Clinical stage**	I/II<br>III/IV	42 / 14	1.000	40 / 12	0.720	34 / 7	1.000	22 / 8	0.649
**Histological subtypes**	NMM<br>SSM<br>Others	47 / 15	0.499	48 / 16	0.592	48 / 13	0.727	30 / 13	0.318
**BRAF status**	Mutated<br>Non-mutated	55 / 18	0.394	56 / 20	0.762	41 / 9	0.360	34 / 15	0.715
**Breslow**	≤1mm<br>>1mm	38 / 12	1.000	37 / 12	0.659	33 / 9	1.000	22 / 11	0.611
**Ulceration**	Yes<br>No	33 / 11	0.721	31 / 8	1.000	27 / 7	1.000	17 / 8	0.347
**Regression**	Yes<br>No	31 / 10	0.967	27 / 7	1.000	25 / 6	1.000	16 / 8	0.200
